# The Phosphonate Derivative of C_60_ Fullerene Induces Differentiation towards the Myogenic Lineage in Human Adipose-Derived Mesenchymal Stem Cells

**DOI:** 10.3390/ijms22179284

**Published:** 2021-08-27

**Authors:** Svetlana V. Kostyuk, Elena V. Proskurnina, Elizaveta S. Ershova, Larisa V. Kameneva, Elena M. Malinovskaya, Ekaterina A. Savinova, Vasilina A. Sergeeva, Pavel E. Umriukhin, Olga A. Dolgikh, Ekaterina A. Khakina, Olga A. Kraevaya, Pavel A. Troshin, Sergey I. Kutsev, Natalia N. Veiko

**Affiliations:** 1 Research Centre for Medical Genetics, ul. Moskvorechye 1, 115522 Moscow, Russia; svet-vk@yandex.ru (S.V.K.); es-ershova@rambler.ru (E.S.E.); kamlar@med-gen.ru (L.V.K.); tigerilina@mail.ru (E.M.M.); savinova.ekaterina96@yandex.ru (E.A.S.); tracytheplane@gmail.com (V.A.S.); pavelum@mail.ru (P.E.U.); dolgiko@med-gen.ru (O.A.D.); kutsev@mail.ru (S.I.K.); satelit32006@yandex.ru (N.N.V.); 2 Department of Normal Physiology, I.M. Sechenov First Moscow State Medical University (Sechenov University) , Mohovaya Str. 11-4, 125009 Moscow, Russia; 3 A.N. Nesmeyanov Institute of Organoelement Compounds of Russian Academy of Sciences, Vavylova St. 28, B-334, 119991 Moscow, Russia; eka57671232@yandex.ru; 4 Institute of Problems of Chemical Physics of Russian Academy of Sciences, Semenov Prospect 1, 142432 Chernogolovka (Moscow Region), Russia; okraevaya@inbox.ru (O.A.K.); troshin2003@inbox.ru (P.A.T.)

**Keywords:** mesenchymal stem cells, fullerene phosphonate derivatives, myogenic differentiation, autophagy, reactive oxygen species

## Abstract

Inductors of myogenic stem cell differentiation attract attention, as they can be used to treat myodystrophies and post-traumatic injuries. Functionalization of fullerenes makes it possible to obtain water-soluble derivatives with targeted biochemical activity. This study examined the effects of the phosphonate C_60_ fullerene derivatives on the expression of myogenic transcription factors and myogenic differentiation of human mesenchymal stem cells (MSCs). Uptake of the phosphonate C_60_ fullerene derivatives in human MSCs, intracellular ROS visualization, superoxide scavenging potential, and the expression of myogenic, adipogenic, and osteogenic differentiation genes were studied. The prolonged MSC incubation (within 7–14 days) with the C_60_ pentaphoshonate potassium salt promoted their differentiation towards the myogenic lineage. The transcription factors and gene expressions determining myogenic differentiation (*MYOD1*, *MYOG*, *MYF5*, and *MRF4*) increased, while the expression of osteogenic differentiation factors (*BMP2*, *BMP4*, *RUNX2*, *SPP1*, and *OCN*) and adipogenic differentiation factors (*CEBPB*, *LPL*, and *AP2* (*FABP4*)) was reduced or did not change. The stimulation of autophagy may be one of the factors contributing to the increased expression of myogenic differentiation genes in MSCs. Autophagy may be caused by intracellular alkalosis and/or short-term intracellular oxidative stress.

## 1. Introduction

Recovery of muscle tissue damaged by diseases or injuries is a complex process. If a massive amount of muscle tissue is lost, the intrinsic regeneration potential of skeletal muscles cannot provide the complete healing of the lesion [1]. Moreover, muscle weakness, muscular dystrophy, and impaired physical mobility are characteristic of genetically heterogeneous myodystrophies such as Duchenne muscular dystrophy, facioscapulohumeral muscular dystrophy, and myotonic dystrophy [2]. Pathogenic variations in the dystrophin gene underlie Duchenne muscular dystrophy. This disease is the most common childhood muscular dystrophy, affecting 1:5000 live male births [3]. Facioscapulohumeral muscular dystrophy is the second most prevalent dystrophy after Duchenne muscular dystrophy, and affects approximately 870,000 people worldwide [4]. Facioscapulohumeral muscular dystrophy results from the inappropriate expression of a gene called Double Homeobox 4 (*DUX4*) [5]. Congenital myotonic dystrophy is an autosomal dominant genetic disorder with an incidence of approx. 1:48,000 live births [6]. There is no treatment for these diseases. Today, methods aimed at the symptomatic correction are physiotherapy and administration of steroids, anticonvulsants, and immunosuppressant drugs. Cellular therapy is a perspective for substituting damaged tissue that makes stem cells a promising instrument for regenerative treatment of myodystrophies and post-traumatic injuries. Mesenchymal stem cells (MSCs) can be attractive for this purpose due to the simplicity of isolation, high migration capacity, relatively high expansion rate, and few allogeneic reactions caused by transplantation [7,8].

**Figure 1:**
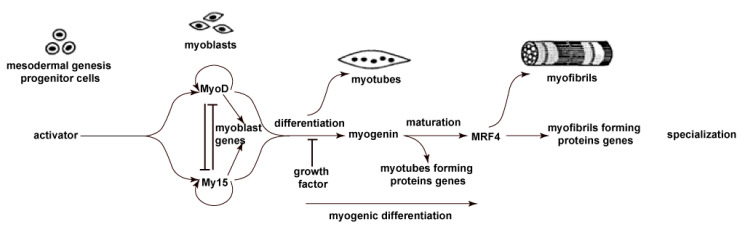
Transcription factors of myogenic differentiation and activation sequence. Reproduced with permission from Olson et al. [34]. Copyright 1994 Author(s).

Myogenic MSC differentiation is caused by activating the sequential expression of transcription of specific myogenic transcription factors such as MYOD, MYF5, myogenin, and MRF4 (Figure 1) [9,10,11], which are also involved in post-traumatic muscle tissue repair [11]. The progression of activated progenitor cells towards the myogenic lineage in the early stages is mainly controlled by the expression of the *MYF5* and *MYOD* genes [12]. The *MYF5* gene is activated in the early differentiation period; however, the mRNA level can remain elevated during the entire differentiation process, decreasing only in the late period [10,13]. *MYF5* is considered a key gene for the initiation of myogenic differentiation [12,14,15,16]. Expression of the *MYOD* gene is initiated shortly after an increase in the *MYF5* gene transcription [17,18]. In proliferating myoblasts, the *MYOD* gene is transcribed through a TFIID-dependent mechanism, in which a moderate level of expression is provided due to spatial localization near the nuclear periphery [19,20]. As myoblasts initiate differentiation towards the myotubes, the *MYOD* gene moves into the nucleus, where the TAF3/TRF3-dependent transcription mechanism leads to a high-level *MYOD* expression [19,20]. MYOD controls the early gene expression in differentiation: adhesion and cell-matrix molecules, intermediate transcription factor genes, and late genes encoding cytoskeletal proteins [21]. MYOD activates transcription activity in previously “silent” loci, acting as a “master switch” gene, which contributes to the transformation of cells of different lines and differentiation stages into skeletal muscle cells [21]. It was shown that there are functional differences inherent in members of the MRF family and their specific spatial and temporal expression patterns during development [14]. The genomewide binding analysis demonstrated that *MYOD* and *MYF5* share a significant proportion of their targets in proliferating myoblasts [22]. Cross-interactions between the *MYOD* and *MYF5* genes also differ in humans and mice. Knockdown of *MYOD1* in human skeletal muscle stem cells does not affect *MYF5* expression. However, *MYF5* directly or indirectly enhances *MYOD1* expression [13]. In murine stem cells, knockdown of *MYOD1* leads to an increase in the expression of the MYF5 protein [23].

An increased MYOD expression stimulates the MYOG expression, which leads to lowering MYF5 expression [24]. This transition of expression from MYF5 to MYOG coincides with the exit from the cell cycle and the exit of the cell into a differentiated state [14,25,26]. MYOD and MYF5 are usually considered factors involved in identifying myogenic cells, while MYOG and MRF4 are more strongly associated with terminal differentiation and myofiber homeostasis [21]. The molecular characterization of two transcription factors, MYOD and MYOG, suggests an essential difference between these factors. MYOD establishes an open chromatin structure in muscle-specific genes, while MYOG provides high levels of gene transcription in this open chromatin state [14,27]. Both in vitro and in vivo, myogenin expression precedes the terminal differentiation of stem cells and controls the synthesis of proteins that make up the contractile apparatus of cells [28].

The joint activity of MYOD and MYOG leads to the expression of the *MRF4* gene and other genes of late muscle differentiation, which allows the formation of multinucleated fibers. In mature muscle fibers, the expression of *MYOD* and *MYOG* decreases, while *MRF4* continues to be expressed at high levels, acting as the predominant MRF in adult muscle [29]. MRF4 is a marker of the late period of muscle differentiation. MRF4 is responsible for the terminal differentiation of muscle fibers [30].

Various biochemical pathways regulate myogenic differentiation. The growth factors TGF-β and FGF, and transcription factors STAT2 and STAT3, are involved in regulating myogenic differentiation [31,32]. Autophagy is another regulator of myogenic differentiation in MSCs [33]. Autophagy is mediated by the *LC3* and *BECN1* genes [33].

Functionalized fullerenes have biochemical effects on stem cells. Fullerenol/alginate hydrogel can effectively decrease the reactive oxygen species level in the myocardial infarction zone, improve the retention and survival of implanted brown-adipose-derived stem cells, and induce angiogenesis. Moreover, fullerenol can improve the cardiomyogenic differentiation of brown-adipose-derived stem cells [35]. Fullerene C_60_ improves the MAPK expression level and brown-adipose-derived stem cell survival and proliferation, and modulates cardiomyogenic differentiation. In addition, fullerene C_60_ promotes the formation of a gap junction among cells [36]. The anti-aging effect of fullerenol on skin aging through derived stem cells in a mouse model was demonstrated by Li et al. [37]. Fullerenol inhibited the crosstalk between bone-marrow-derived mesenchymal stem cells and tumor cells by regulating MAPK signaling [38].

Thus, studies on the effects of fullerenes and their derivatives on the proliferation and differentiation of stem cells are very promising, but not numerous. We have not been able to find information on water-soluble fullerenes as inducers of myogenic differentiation in stem cells. We have previously demonstrated the increased expression of some myogenic differentiation factors in MSCs by a water-soluble phosphonate C_60_ derivative [39]. Here, we have studied in detail the effect of this compound compared to two other phosphorus-containing compounds on autophagy and the expression of myogenic, adipogenic, and osteogenic transcription factors in human adipose-derived MSCs.

## 2. Results

**Figure 2:**
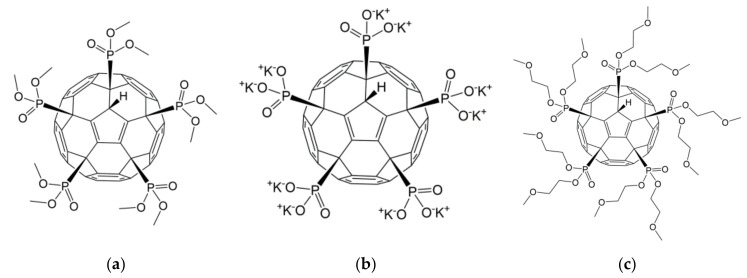
The molecular formula of the phosphonate derivatives of C_60_ fullerene F1 (**a**), F2 (**b**), and F3 (**c**).

The effect of three phosphorus-containing water-soluble derivatives of C_60_ fullerene on MSCs obtained from different donors was studied. The molecular structures of the fullerenes (F1, F2, and F3) are shown in Figure 2.

### 2.1. Permeation and Distribution of Fullerenes in Cells

The standard MTT test was performed to assess the cytotoxicity of the fullerenes. The maximum nontoxic concentrations were as high as 175 µM for F1, 200 µM for F2, and 295 µM for F3.

**Figure 3:**
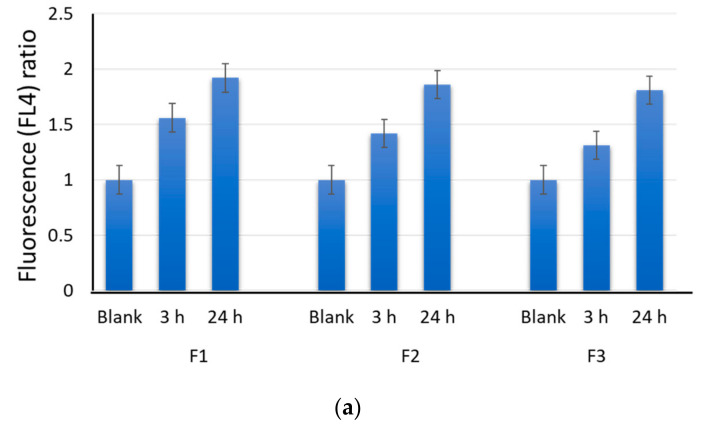

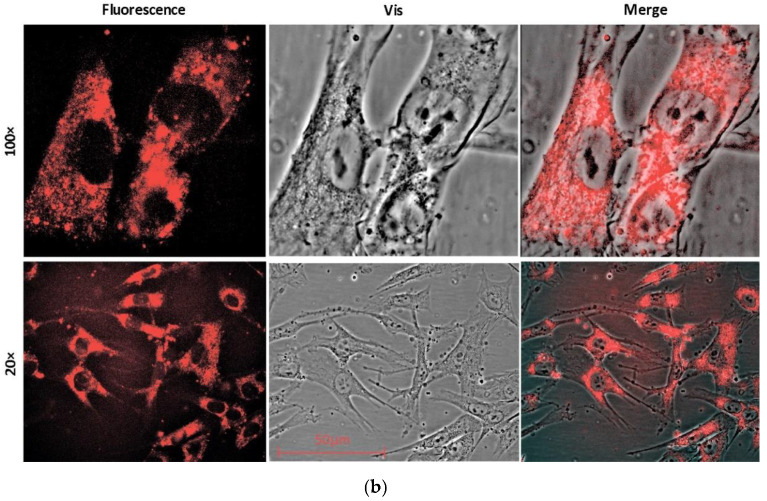
Permeation of F1 (170 µM), F2 (195 µM), and F3 (290 µM) into MSCs after 3 h and 24 h of incubation assessed with flow cytometry. The fluorescence (FL4) is the mean value for three experiments related to the blank (**a**) and distribution of F2 (195 µM) in the cells after 24 h of incubation detected with fluorescence microscopy (20× and 100×) (**b**).

We have previously shown that aqueous solutions of C_60_ derivatives exhibit dark-red fluorescence in the range of 600–950 nm excited by a wide wavelength range (from 300 to 600 nm) [40,41,42], which makes it possible to analyze their distribution in cells. Within 3–24 h after adding to the cells, F1 (170 µM), F2 (195 µM), and F3 (290 µM) permeated into MSCs as the fluorescence intensity increased by a factor of 1.5–2 (Figure 3a). The maximum fluorescence was detected in 24 h. A longer incubation time did not significantly influence the permeation of the fullerenes into the cells.

The intracellular distribution of the fullerenes was visualized as dark-red fluorescence (λ_ex_ = 350 nm). Figure 3b shows the fluorescence image of F2 localizing in the cells after 24 h of incubation.

### 2.2. F1, F2, and F3 Stimulate the Expression of Transcription Factors of Myogenic Differentiation in MSCs to Varying Degrees

We examined the effect of water-soluble C_60_ fullerene derivatives F1, F2, and F3 in two concentrations on the expression of both genes and proteins. F1 was taken at concentrations of 398 nM and 170 μM; F2 was taken at concentrations of 456 nM and 195 µM; and F3 was taken at concentrations of 679 nM and 290 µM. The micromolar concentrations were chosen based on the MTT test. These are the maximum nontoxic concentrations for stem cells. The nanomolar concentrations were 427 times lower than the maximum nontoxic concentrations of the studied substances. The fullerenes at these concentrations still affected the gene expression. At lower concentrations, there was no effect on the cells.

#### 2.2.1. MYF5

**Figure 4:**
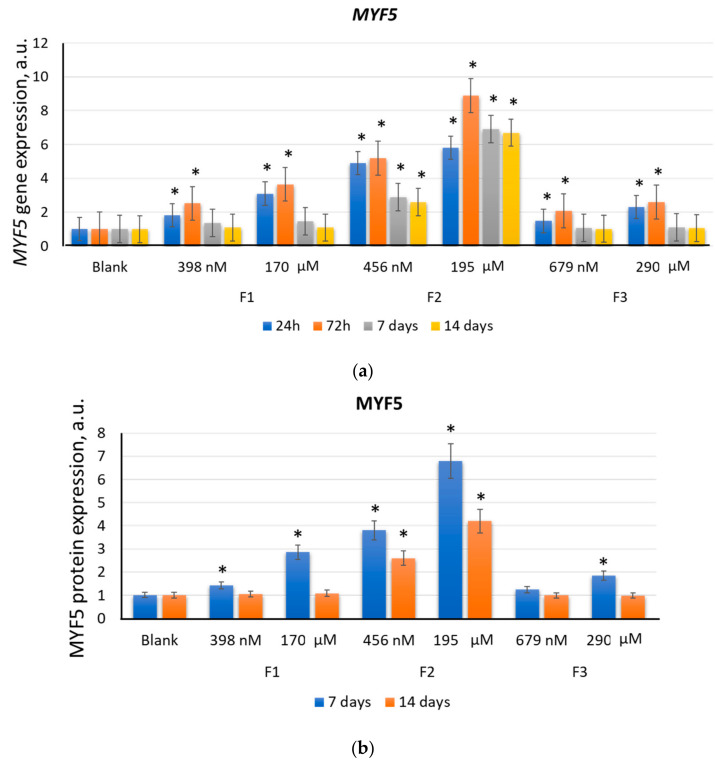
The effect of F1, F2, and F3 on the *MYF5* gene expression (**a**) and MYF5 protein expression (**b**); the concentrations and exposition times are shown in the figure. The gene RNA amount was the mean value for three experiments related to the blank; the *TBP* gene was used as an internal reference gene; (*) denotes significant differences compared to blank cells, *p* < 0.005, in a Mann–Whitney test. In blank experiments, cells were incubated without the fullerenes.

Each of the three tested compounds activated *MYF5* gene expression to varying degrees. F3 at concentrations of 679 nM and 290 μM stimulated the expression of the *MYF5* gene by a factor of 1.8–2.3 (*p* < 0.01) after 24–72 h of incubation. After 7 days, the *MYF5* expression decreased to the blank level (Figure 4a). F1 at concentrations of 398 nM and 170 μM stimulated the expression of the *MYF5* gene by a factor of 3–3.7 (*p* < 0.01) and 1.8–2.3 (*p* < 0.01), respectively, after 24–72 h of incubation. After 7 days, the *MYF5* expression remained 1.5 times higher than the blank value (*p* < 0.01). After 14 days, the *MYF5* expression returned to the blank value. F2 caused the most significant changes in the transcriptional activity of the *MYF5* gene. After 24–72 h of incubation with 456 nM, the *MYF5* expression increased by a factor of 4.8–5.3 (*p* < 0.01). After 24–72 h of incubation with 195 μM, the *MYF5* expression increased by a factor of 6.0–9.0 (*p* < 0.01). After 7 and 14 days, the *MYF5* expression remained increased by a factor of 2.6–2.9 (*p* < 0.01) for 456 nM and 6.7–6.9 (*p* < 0.01) for 195 µM (Figure 4a).

The activity of *MYF5* gene increases in the early differentiation period; however, the mRNA level can remain elevated during the entire differentiation process, decreasing only in the late period [10,13]. Some researchers have reported a decrease in the *MYF5* protein expression during cell-cycle progression due to proteolytic degradation [43,44], associated with the termination of proliferative signals. However, F2, in contrast to F1 and F3, activates *MYF5* transcription in MSCs for a long period of time, which contributes to the prolonged differentiation and an increase in the expression of the MYF5 transcription factor. As a result of the activation of the *MYF5* transcriptional activity, rapid translation of *MYF5* mRNA occurs [45].

In line with the above, an increase in the *MYF5* gene expression led to increased *MYF5* protein expression. After 7 days of incubation with F1 at concentrations of 398 nM and 170 μM or F3 at a concentration of 290 μM, the MYF5 protein expression increased by a factor of 1.5–2.9 (*p* < 0.01). After 14 days, the MYF5 protein expression decreased to the blank level (Figure 4b). After 7–14 days of incubation with F2 at concentrations of 456 nM and 195 µM, the MYF5 protein expression increased by a factor of 2.5–4.0 (*p* < 0.01) and 4.0–7.0 (*p* < 0.01), respectively (Figure 4b).

#### 2.2.2. MYOD

**Figure 5:**
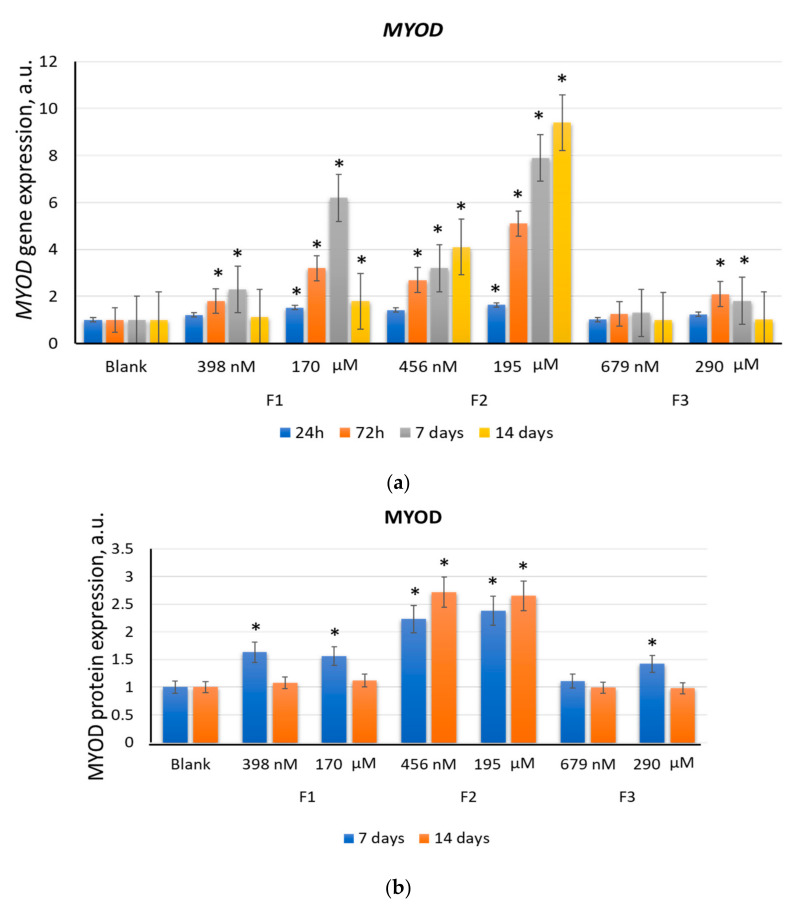
The effect of F1, F2, and F3 on the *MYOD1* gene expression (**a**) and MYOD1 protein expression (**b**); the concentrations and exposition times are shown in the figure. The gene RNA amount was the mean value for three experiments related to the blank; the *TBP* gene was used as an internal reference gene; (*) denotes significant differences compared to blank cells, *p* < 0.005, in a Mann–Whitney test. In blank experiments, cells were incubated without the fullerenes.

All three tested compounds stimulated the *MYOD1* gene expression to varying degrees. After 3–7 days of incubation with F3 (290 μM), the expression level of the *MYOD1* gene increased by a factor of 1.5–2.0 (*p* < 0.01). After 14 days, the *MYOD1* gene expression decreased to the blank values. At a concentration of 679 nM, F3 did not significantly affect the *MYOD1* gene expression (Figure 5a).

After 24 h, 72 h, and 7 days of incubation with F1 (170 μM), the *MYOD1* gene expression increased by a factor of 1.5, 3.2, and 6.2 (*p* < 0.01 in all cases), respectively. After 14 days, the *MYOD1* gene expression remained 1.5 times higher than the blank value (*p* < 0.01) (Figure 5a). After 3–7 days of incubation with F1 (398 nM), the *MYOD1* gene expression increased by a factor of 1.5–2.3 (*p* < 0.01). After 14 days, the *MYOD1* gene expression decreased to the blank values (Figure 5a).

The transcriptional activity of the *MYOD1* gene changed most significantly under the influence of F2. After 3–14 days of incubation with F2 (456 nM), the *MYOD1* gene expression increased by a factor of 2.7–4.1 (*p* < 0.01). As a result of incubation with 195 μM F2, the *MYOD1* gene expression increased significantly after 24 h. After 3, 7, and 14 days, the *MYOD1* expression increased with time, exceeding the blank values by a factor of 5, 8, and 9.5 (*p* < 0.01 in all cases), respectively (Figure 5a).

The MYOD1 protein expression increased by a factor of 1.5–2.2 (*p* < 0.01) after 7 days of incubation with F1 (170 μM and 398 nM) and F3 (290 μM). After 14 days, the MYOD1 protein expression decreased to the blank values (Figure 5b). As for F2 (456 nM and 195 μM), the MYOD1 protein expression increased by a factor of 2.2–2.8 (*p* < 0.01) after 7–14 days of incubation (Figure 5b).

Thus, F2 activated the *MYOD1* gene for two weeks, which allowed it to bind to regulatory elements expressed at the early and late stages of differentiation [34]. Our results demonstrate a long-term increased level of *MYOD1* mRNA during differentiation in the presence of F2, which coincided with the previously obtained data [34,46].

The MYOD protein expression was not significantly increased after 1–4 days of incubation with F1 (398 nM and 170 µM) (Figure 5b), while the *MYOD1* gene expression was high. This phenomenon possibly resulted from the fast degradation of the protein, which was in agreement with the previously obtained data [10,47]. Degradation of MYOD may be associated with cell-cycle progression [48] and regulated by lysine 133, which plays a crucial role in the ubiquitination and degradation of MYOD [48].

#### 2.2.3. MYOG

**Figure 6:**
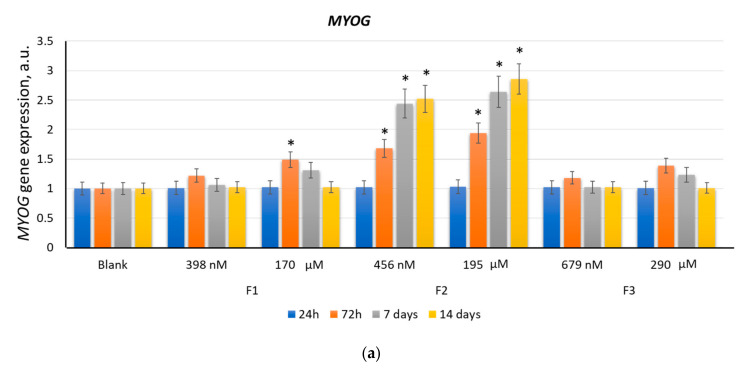

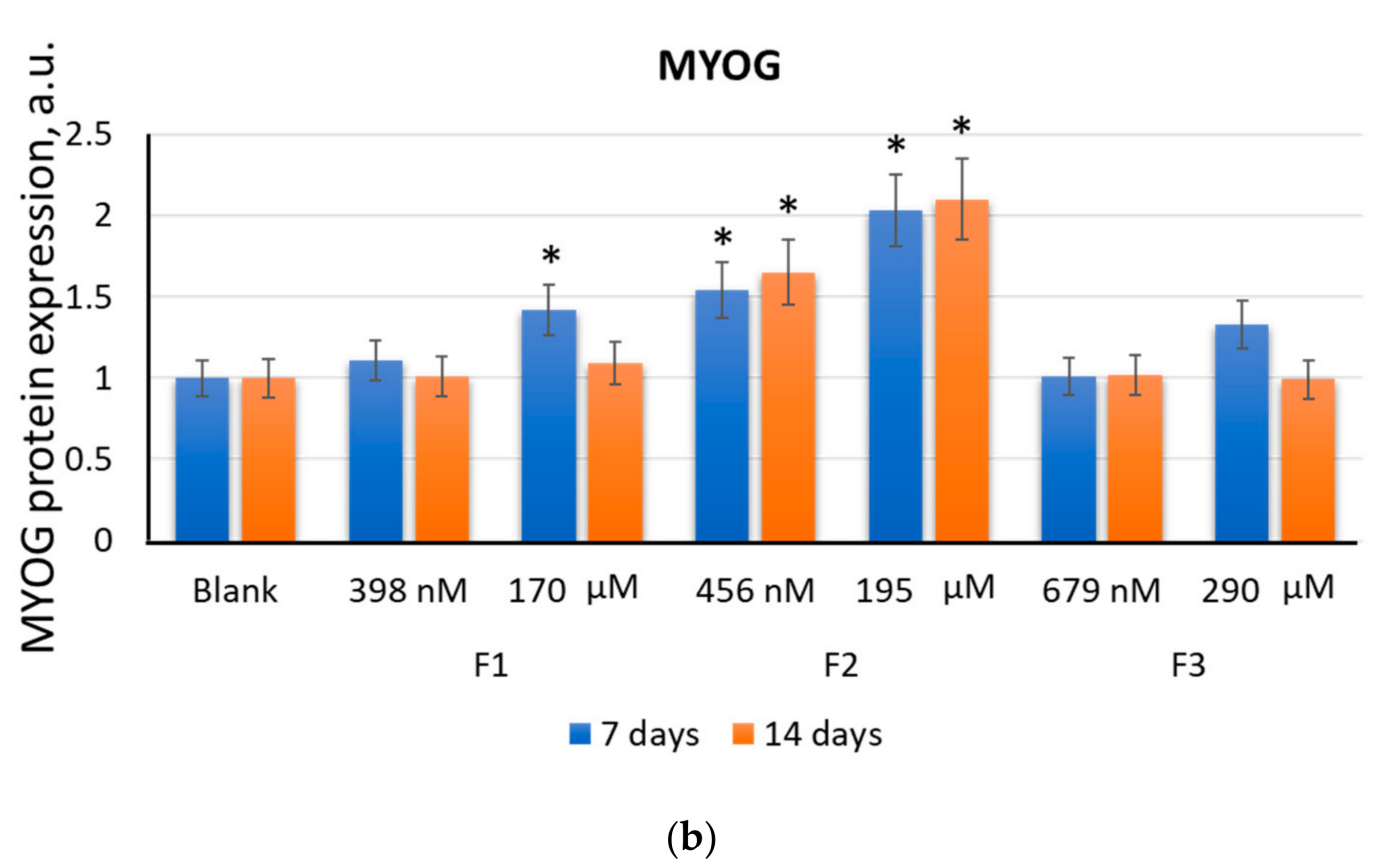
The effect of F1, F2, and F3 on the *MYOG* gene expression (**a**) and MYOG protein expression (**b**); the concentrations and exposition times are shown in the figure. The gene RNA amount was the mean value for three experiments related to the blank; the *TBP* gene was used as an internal reference gene; (*) denotes significant differences compared to blank cells, *p* < 0.005, in a Mann–Whitney test. In blank experiments, cells were incubated without the fullerenes.

The *MYOG* gene expression increased approximately 1.5 times (*p* < 0.01) after 3 days of incubation with F1 (170 µM). After 7 days, the *MYOG* gene expression decreased, reaching the blank level after 14 days (Figure 6a). F3 (679 nM and 290 µM) and F1 (398 nM) did not cause a statistically significant increase in the *MYOG* gene expression (Figure 6a). Unlike F1 and F3, F2 (456 nM and 195 μM) significantly affected the *MYOG* gene expression. After 3–14 days, the *MYOG* gene expression increased by a factor of 1.9–2.9 (*p* < 0.01) (Figure 6a).

The MYOG protein expression increased approximately 1.5 times (*p* < 0.01) after 7 days of incubation with F1 (170 μM). After 14 days, the MYOG protein expression decreased to the blank values (Figure 6b). F3 (679 nM and 290 µM) and F1 (398 nM) did not significantly affect the MYOG protein expression (Figure 6b).

F2 (456 nM and 195 μM) stimulated the MYOG protein expression by a factor of 1.5–2.2 (*p* < 0.01) (Figure 6b). Myogenin plays a crucial role in myogenesis in mice; however, in humans, there are compensation mechanisms that promote the myogenic differentiation of stem cells even in the absence of *myogenin* expression [49,50].

#### 2.2.4. MRF4

**Figure 7:**
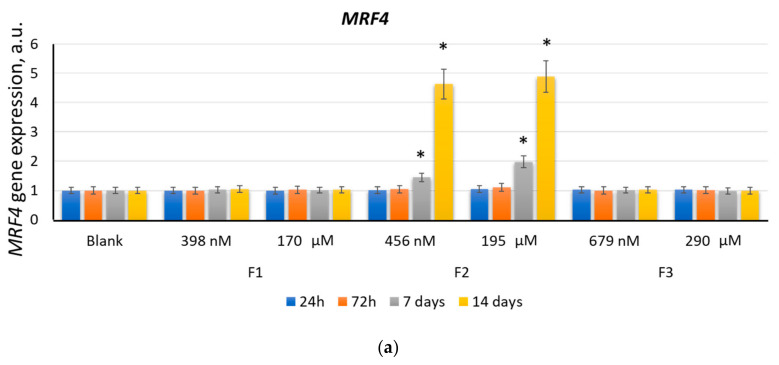

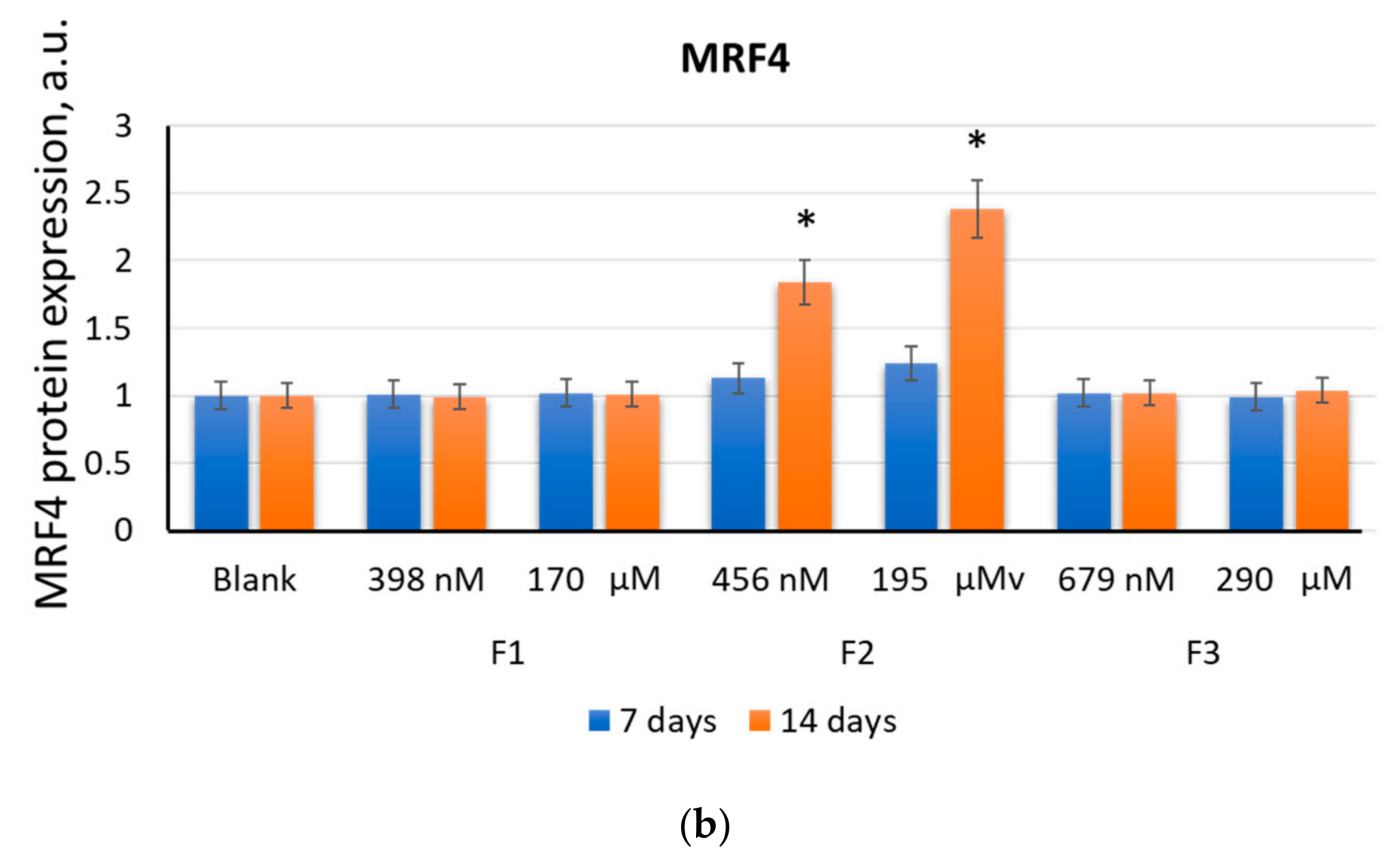
The effect of F1, F2, and F3 on the *MRF4* gene expression (**a**) and MRF4 protein expression (**b**); the concentrations and exposition times are shown in the figure. The gene RNA amount was the mean value for three experiments related to the blank; the *TBP* gene was used as an internal reference gene; (*) denotes significant differences compared to blank cells, *p* < 0.005, in a Mann–Whitney test. In blank experiments, cells were incubated without the fullerenes.

The *MRF4* gene expression increased after 7 days of incubation with F2 (456 nM and 195 μM) by a factor of 1.5–2.0 (*p* < 0.01). After 14 days, the *MRF4* gene expression increased by a factor of 4.6–5.0 (Figure 7a). F1 and F3 did not affect the *MRF4* gene expression (Figure 7a). The MRF4 protein expression increased by a factor of 1.8–2.4 after 14 days of incubation with F2 (456 nM and 195 µM) (Figure 7b).

#### 2.2.5. Immunohistochemical Staining

**Figure 8:**
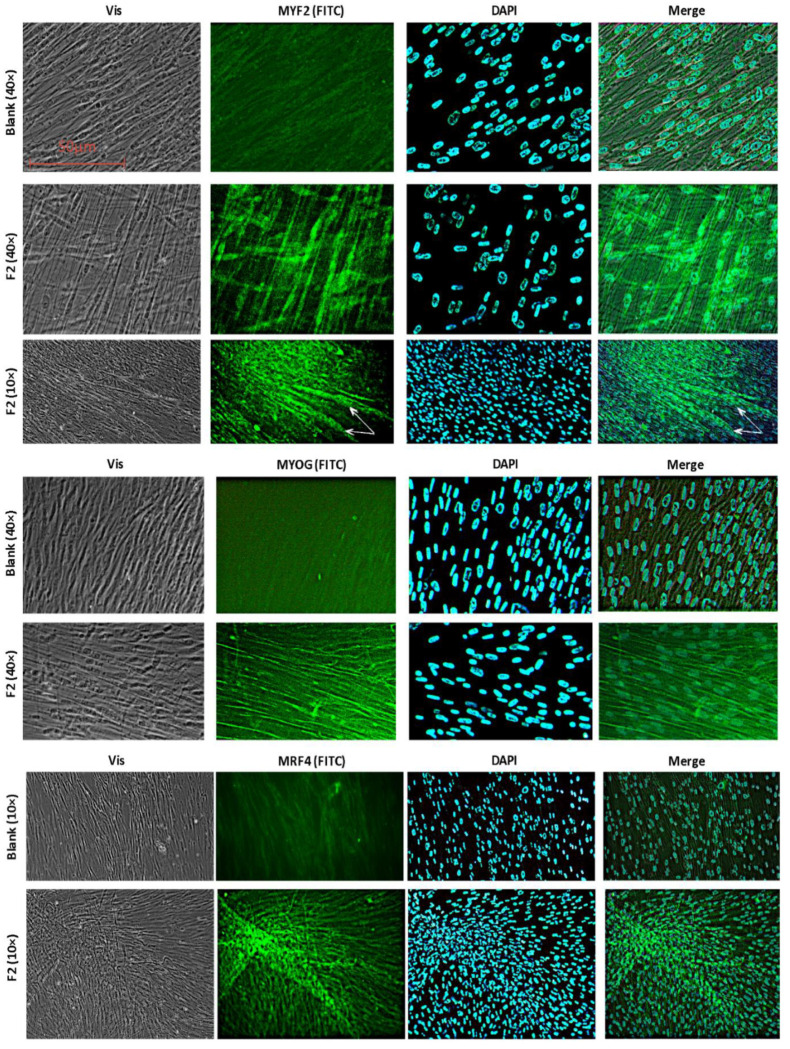
Expression and distribution of the transcription factors of myogenic differentiation 14 days after exposure to fullerene F2 (195 μM). Fluorescence microscopy, 40×, from left to right: visible light, fluorescence image of cells treated with antibodies to the proteins, DAPI staining of nuclei, a merged photo, merged fluorescence, and a transmitted light brightfield images photo.

Immunohistochemical staining of MSCs with antibodies to *MYF5* and *MYOG* after 14 days of incubation with F2 (195 μM), which induced the transcriptional activity of these genes the most, demonstrated an increase in the expression of MYF5 and MYOG proteins in the nuclei and cytoplasm of cells (Figure 8). Analysis of immunohistochemical staining of MSCs with antibodies to MRF4 after 14 days of incubation with F2 (195 μM) showed increased expression of the MYF5 protein in several cells that formed myotube clusters (Figure 8), which indicates the formation of muscle tissue [33].

Thus, F2 in the concentration range of 456 nM–195 μM stimulated the differentiation towards the myogenic lineage in human mesenchymal stem cells. The two other studied phosphorus-containing compounds also stimulated the transcriptional activity of myogenic differentiation genes, but they could not promote the differentiation of stem cells to the terminal stage. Despite the activation of transcription and translation of the *MYF5* and *MYOD* genes by F1 and F3, these compounds did not activate the transcriptional activity of the *MYOG* gene, which led to no effect on the expression of the *MRF4* gene and, apparently, other genes of late muscle differentiation.

### 2.3. Effects of F1, F2, and F3 on the Expression of Genes of the Osteogenic and Adipogenic Differentiation

Since F2 stimulated the expression of main genes of myogenic differentiation, and F1 and F3 activated the transcription of genes for initial myogenic differentiation, we studied the expression of genes for adipogenic and osteogenic differentiation.

**Figure 9:**
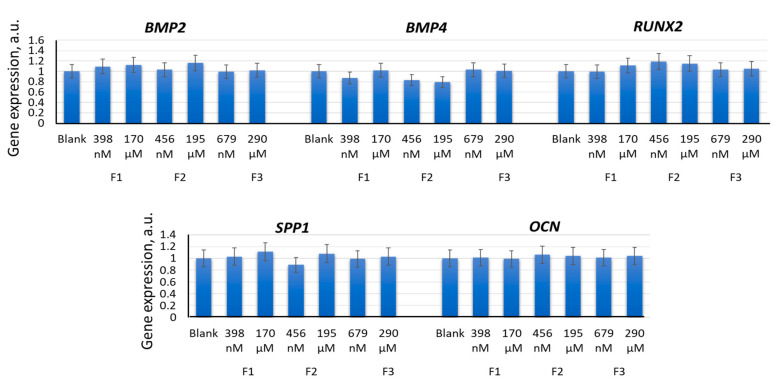
The effect of F1, F2, and F3 on the osteogenic differentiation genes; the concentrations and exposition times are shown in the figure. The gene RNA amount was the mean value for three experiments related to the blank; the *TBP* gene was used as an internal reference gene. In blank experiments, cells were incubated without the fullerenes.

**Figure 10:**
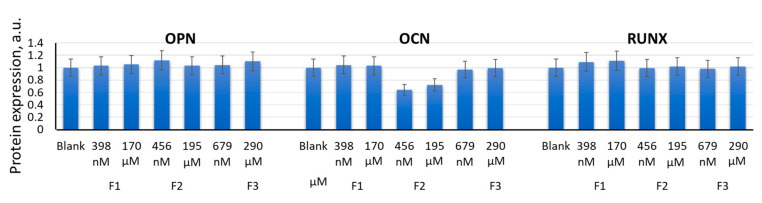
The effect of F1, F2, and F3 on the osteogenic differentiation proteins; the concentrations are shown in the figure. FL1 values were calculated for three experiments related to the blank. In blank experiments, cells were incubated without the fullerenes.

After 14 days of incubation of MSCs with F1 (398 nM and 170 µM), F2 (456 nM and 195 μM), and F3 (679 nM and 290 μM), there was no activation of the expression of either marker genes for osteogenic differentiation (*BMP2*, *BMP4*, *RUNX2*, *SPP1*, and *OCN* [9]) or transcription factors (Figure 9). Osteocalcin tended to decrease after 14 days of incubation with F2 (456 nM and 195 μM) (Figure 10). These data demonstrated a reduced osteogenic potential of MSCs in the presence of F2 (195 µM) within 14 days of incubation.

**Figure 11:**
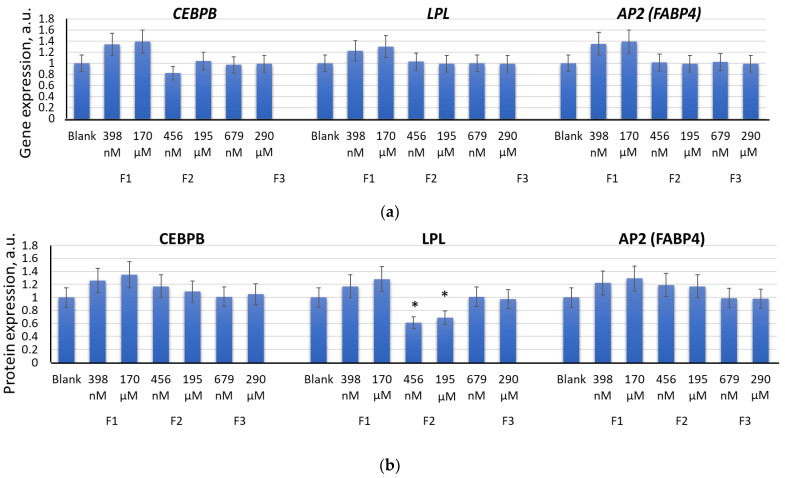
The effect of F1, F2, and F3 on the adipogenic differentiation genes (**a**) and proteins (**b**); the concentrations of fullerenes are shown in the figure. The gene RNA amount was the mean value for three experiments related to the blank; the *TBP* gene was used as an internal reference gene; FL1 values were calculated for three experiments related to the blank; (*) denotes significant differences compared to blank cells, *p* < 0.005, in a Mann–Whitney test. In blank experiments, cells were incubated without the fullerenes.

Incubation of MSCs with F1 (398 nM and 170 μM), F2 (456 nM and 195 μM), or F3 (679 nM and 290 μM) for 14 days did not result in significant activation of expression of either the adipogenic differentiation genes *CEBPB*, *LPL*, and *AP2* (*FABP4*) [9] or the respective proteins (Figure 11a,b). After 14 days, incubation with F1 (398 nM and 170 μM) resulted in a slight increase in the expression of *CEBPB*, *LPL*, and *AP2* (*FABP4*) genes, which may indicate either a switch of myogenic differentiation towards an adipogenic one or activation of adipogenic differentiation in a fraction of the MSC population. Fourteen days of incubation with F2 (456 nM and 195 μM) resulted in a significant decrease in the FABP4 protein, in parallel with an increase in the MYOD protein (Figure 5b).

### 2.4. Effects of F1, F2, and F3 on Intracellular Reactive Oxygen Species in MSCs

H_2_O_2_ and other reactive oxygen species (ROS) are of great importance in the functioning of cells. ROS at low concentrations regulate the cell cycle, protein kinase activity, and gene expression. An increase in ROS synthesis leads to the development of oxidative stress in cells [46]. Effective antioxidants can reduce the delay in stem-cell differentiation in the myogenic direction caused by oxidative stress by removing reactive oxygen species [51].

To study the effects of F1, F2, and F3 on intracellular ROS, we used H2DCFH-DA (2,7-dichlorofluorescein diacetate or 2,7-dichlorodihydrofluorescein diacetate), which permeates through the cell membrane and undergoes deacetylation by intracellular esterases [52]. In the cytosol, nonfluorescent dichlorodihydrofluorescein (DCFH) is a sensitive intracellular marker of oxidative stress during its oxidation to dichlorofluorescein (DCF) by various ROS radicals; DCFH oxidation is nonspecific, and the DCF signal in cells reflects the overall amount of ROS [53]. ROS was detected by flow cytometry assay.

**Figure 12:**
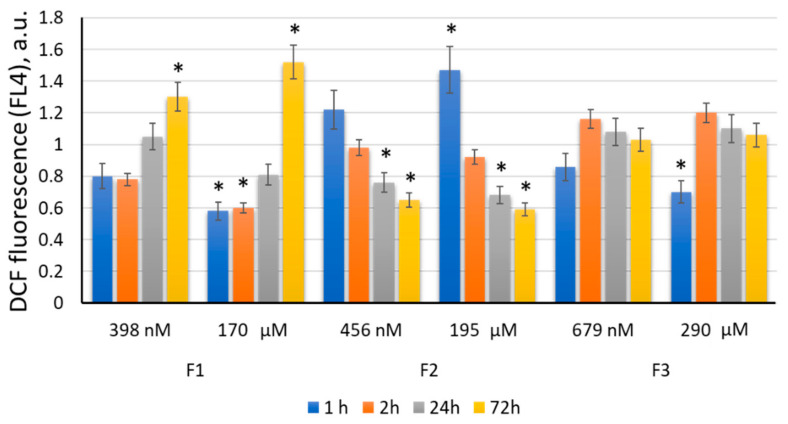
ROS levels in cells expressed as a ratio of DCF synthesis rate constants after F2 (195 μM) exposure related to blank cells (the cells cultured without the fullerene); (*) denotes significant differences according to the Mann–Whitney test (*p* < 0.05). In blank experiments, cells were incubated without the fullerenes.

After 1 to 3 h of incubation with F1 (170 μM), the ROS amount decreased significantly, by 40–42% (*p* < 0.01). After 24 h, the ROS level remained lowered; after 72 h, it increased by 43–47% (*p* < 0.01). A similar but less marked tendency was found for F1 at a concentration of 398 nM (Figure 12).

After 1 h of incubation with F2 (195 μM), the ROS amount increased by 40–45% (*p* < 0.01). After 3 h, the ROS level decreased lower than the blank values. After 24 h, intracellular ROS significantly decreased and remained lowered by 38–42% (*p* < 0.01). A similar but less marked tendency was found for F2 at a concentration of 456 nM. The decrease in ROS as a result of F2 action (456 nM) within 24–72 h was statistically significant (*p* < 0.01) (Figure 12). The decrease in the ROS level in MSCs under F2 action may be associated with its penetration into the MSC cytoplasm after 24 h of incubation.

After 1 h of incubation with F3 (290 μM), there was a significant decrease in the ROS level, by 30–38% (*p* < 0.01). After 3 h, the ROS level increased slightly, and after 24–72 h, it returned to the blank values. A similar but not so marked tendency was observed for F3 at a concentration of 679 nM (Figure 12).

### 2.5. Autophagy Promotes Myogenic Differentiation of MSCs

**Figure 13:**
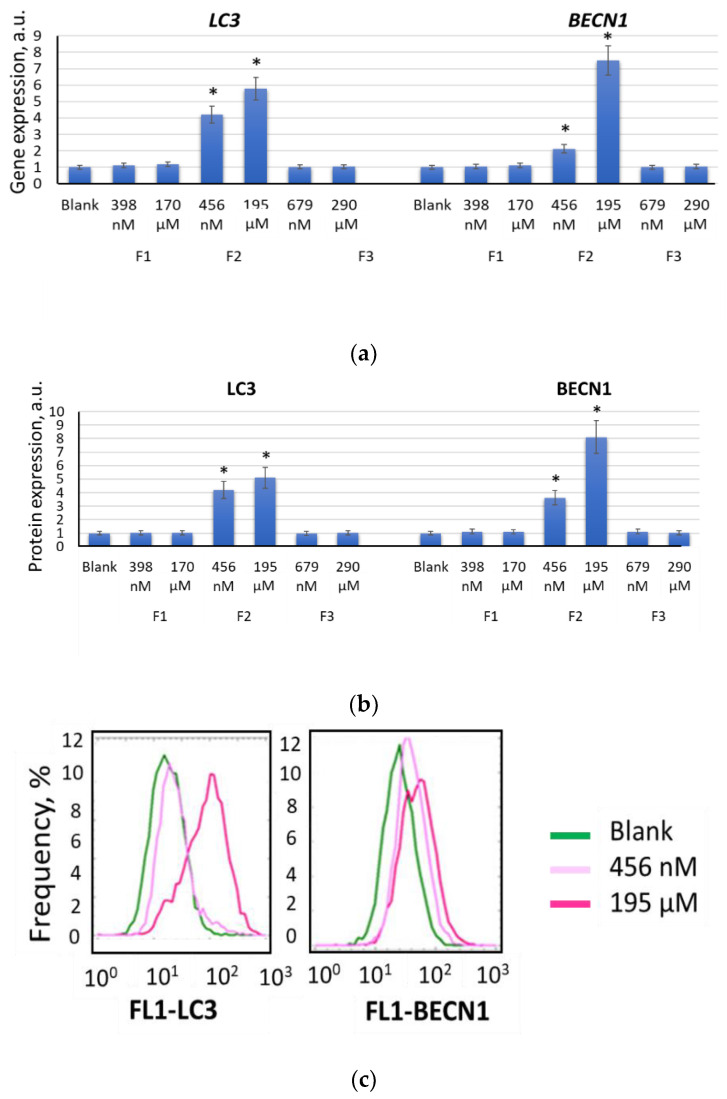

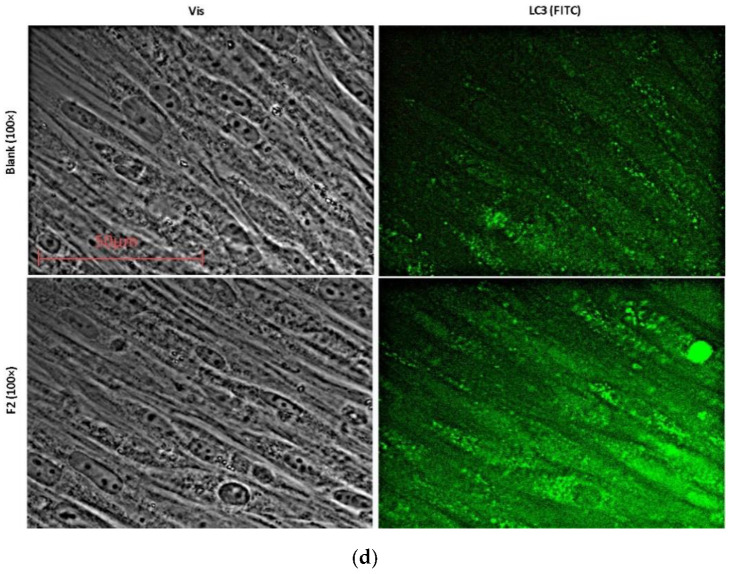
The effect of F1, F2, and F3 on the autophagy genes (**a**) and proteins (**b**); the concentrations of fullerenes are shown in the figure. The gene RNA amount was the mean value for three experiments related to the blank; the *TBP* gene was used as an internal reference gene; FL1 values were calculated for three experiments related to the blank; (*) denotes significant differences compared to blank cells, *p* < 0.005, in a Mann–Whitney test. Distribution of MSCs treated with F2 (195 μM, 14 days) according to the intensity of the antibody signal (**c**). Expression of LC3 (staining with fluorescein isothiocyanate (FITC)) 14 days after incubation of MSCs with F2 (195 μM) (**d**); fluorescence microscopy, 40×. In blank experiments, cells were incubated without the fullerenes.

After 7 days of incubation with F2 (456 nM), the expression of the *LC3* gene and the LC3 protein increased by a factor of 4.0–4.2 (*p* < 0.01), and the *BECN1* gene and BECN1 protein by a factor of 1.5–2.0 (*p* < 0.01) (Figure 13a,b). The activation of the expression of genes and proteins involved in autophagy was even more marked under the action of F2 at a concentration of 195 μM. After 7 days, the expression of the *LC3* gene and the LC3 protein increased by a factor of 5.0–5.8 (*p* < 0.01), and the *BECN1* gene and BECN1 protein by a factor of 7.2–8.0 (*p* < 0.01) (Figure 13a,b). The results were confirmed by immunohistochemistry. When MSCs were stained with antibodies to LC3, the number of LC3-positive cells increased, and the fluorescence of antibodies increased after 14 days of incubation with F2 at a concentration of 195 µM (Figure 13d). Incubation of MSCs with F1 (398 nM and 170 μM) and F3 (679 nM and 290 µM) for 7–14 days did not stimulate the expression of either *LC3* autophagy gene or the LC3 protein (Figure 13c).

Thus, the stimulation of autophagy may be another factor contributing to the activation of the expression of factors of myogenic differentiation in MSCs by F2.

## 3. Discussion

When studying the differentiation of MSCs, we assumed that not all cells in the population could be differentiated under the influence of the fullerenes. Therefore, we used the flow cytometry method, as it provided not only the total amount of proteins, but also the percentage of cells in which protein expression had changed. In addition, the data were confirmed by fluorescence microscopy. The photographs demonstrate that the fluorescence did not increase in all cells. However, the total protein expression significantly increased; this allowed us to consider the stimulus of F2 quite effective.

All studied compounds were fullerenes C_60_, modified with five phosphonic acid residues. They differed from each other in substituents at the P–O bond. Compound F2 was C_60_ pentaphoshonate potassium salt, F1 was pentadimethylphosphonate, and F3 was pentamethoxyethyl phosphonate. Thus, F2 was an acid anion, and the other two compounds were esters. These compounds have comparable toxicity (0.2–0.3 mM), but the biological activity of F2 concerning the differentiation of mesenchymal stem cells differs significantly from that of F1 and F3. While the latter two fullerenes activate only the early genes of myogenic differentiation, and F1 is a slightly more effective stimulus than F3, F2 activates the genes of the entire myogenic differentiation pathway. None of the compounds affected the pathway of osteogenic or adipogenic differentiation. In addition, F2 turned out to be an inducer of autophagy. These results are summarized in Table 1.

**Figure A1:**
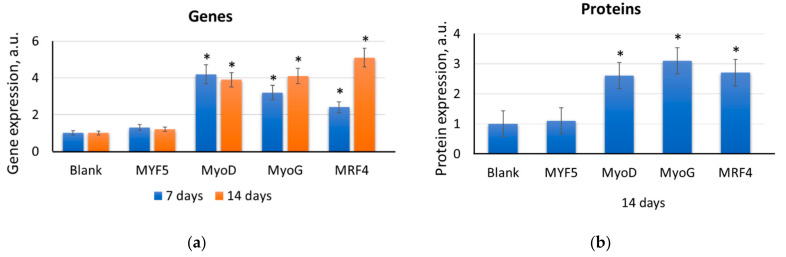
The expression of genes (**a**) and proteins (**b**) of myogenic differentiation induced by the standard differentiation kit (a positive control); the exposition times are shown in the figure. The gene RNA amount was the mean value for three experiments related to the blank; the *TBP* gene was used as an internal reference gene; (*) denotes significant differences compared to blank cells, *p* < 0.005, in a Mann–Whitney test. In blank experiments, cells were incubated without the differentiation kit.

It was essential to compare the effects of F2 fullerene with a conventional inducer of myogenic differentiation. We performed such experiments with a standard myogenic differentiation kit (a positive control). The F2-induced expression of genes and proteins involved in myogenic differentiation was comparable with the positive control. There were minor differences between the expression of early genes of myogenic differentiation. At 7 and 14 days, the expression of MYOD, MYOG, and MRF4 genes and proteins was almost the same for F2 and the positive control, while the expression of MYF5 gene and protein was increased for F2-induced stimulation, but not for the positive control (Figure A1, Table A1). This may be probably due to a different mechanism of activation of the transcriptional activity of genes, which was shown, for example, by Mizuno et al. [54].

Thus, the differentiation stimulated by F2 reached late stages, which opened prospects for further study and searches for similar derivatives with higher differentiation potential. The phenomenon of the induction of differentiation towards the muscle lineage by phosphorous-containing derivatives, in our opinion, can be considered as a new significant fact that requires description and discussion.

Conclusions about all possible mechanisms of triggering autophagy and myogenic differentiation cannot be made without specific experiments. Here, we only discuss the relationship between MSC differentiation, autophagy, and ROS disbalance.

### 3.1. MSC-Based Therapy of Muscular Dystrophies

Adipose-derived MSCs represent an attractive cell type for medical applications, as they can be isolated readily from fat [55,56]. Adipose-derived stem cells can differentiate towards osteogenesis, adipogenesis, and chondrogenesis [55,57]. Their ability to undergo myogenesis, however, appears to be contradictory. MSCs have immunomodulatory/immunoregulatory activity, which is provided both by direct intercellular contacts and due to the expression and secretion of many molecules with immunomodulatory properties [58]. Immunomodulation and secretion of growth factors by MSCs provide the potential of MSC-based cell therapy [59]. The use of MSCs as immunomodulators has been explored in transplantation [60,61], tissue repair [62], treatment of autoimmune diseases [63], asthma [64], prevention of graft vs. host disease, and even for treatment of COVID-19 [65]. The regenerative and reparative potential of MSC-based therapy was developed for the treatment of aging frailty [66], liver cirrhosis [67] and fibrosis [68], cutaneous wounds [69], heart failure [70], ischemic stroke [71], and in many other cases. However, despite these positive results, there is still a problem with the potential of MSCs to promote tumorigenesis [72].

As for muscular dystrophies, in contrast to other cell-based therapies, MSCs have the advantages of (a) the ability to fuse with and genetically complement dystrophic muscle; (b) possessing anti-inflammatory activities; and (c) producing trophic factors that may augment the activity of endogenous repair cells [73]. Goncalves et al. demonstrated that human mesenchymal stem cells ectopically expressing full-length dystrophin could complement Duchenne muscular dystrophy myotubes by cell fusion [74]. Siemionow et al. developed dystrophin-expressing chimeric human cells of myoblast/mesenchymal stem cell origin for transplantation in Duchenne muscular dystrophy [75]. Intramuscular injection of nonautologous MSCs can be safely used to treat dystrophic muscle in immunocompetent hosts without inflaming the host immune system [76]. The review [77] summarizes data on MSC-based therapy for Duchenne muscular dystrophy. Human umbilical cord mesenchymal stem cells were successfully applied in the safe and efficient treatment of Duchenne muscular dystrophy in India [78]. Human adipose-derived CD146(+) stem cells increased the life span of a muscular dystrophy mouse model more efficiently than mesenchymal stromal cells, possibly due to immunomodulatory and angiogenic properties [79]. Administration of microfragmented fat in key muscles improved muscular phenotype (decreased necrosis and fibrosis), decreased inflammatory cytokines, and increased strength in a murine model of Duchenne muscular dystrophy [80].

### 3.2. ROS and Differentiation of MSCs

MSCs are known to have low levels of intracellular ROS and high levels of glutathione. Increasing evidence implicates a tight regulation of differentiation by reactive oxygen species. ROS interact with several differentiation pathways, such as the Wnt, Hedgehog, and FOXO signaling cascades. On the other hand, oxidative stress leads to the arrest of the MSC cell cycle and apoptosis. Tightly regulated levels of ROS are therefore critical for MSC terminal differentiation, although the precise sources, localization, levels, and the exact species of ROS implicated remain to be determined [81].

ROS primarily stimulate the expression of genes associated with adipogenesis and favor human MSC differentiation into mature adipocytes. Moreover, adipocytes contain higher levels of intracellular ROS compared with progenitors [81]. ROS regulate adipocyte differentiation of MSCs by activating peroxisome proliferator-activated receptor gamma (PPARγ,) while the antioxidant N-acetyl-L-cysteine acts through ROS to inhibit adipocyte differentiation [82]. The hypoxia-induced increase in mitochondrial ROS stimulated adipocyte differentiation via the PI3K/Akt/mTOR pathway [83].

Besides mitochondria, NADPH oxidases (NOX) are a major source of ROS. Knockdown of *NOX4* inhibited ROS production and adipocyte differentiation by differentiation-inducing agents. Thus, the increase in the intracellular ROS level via NOX4 mediates adipocyte differentiation in MSC [84].

It is believed that ROS suppress osteoblast differentiation, and antioxidants could potentially neutralize this effect [81]. To prove this statement, the researchers investigated the osteogenic differentiation effect of resveratrol on senescent bone mesenchymal stem cells and the involvement of the AMP-activated protein kinase (AMPK)/(ROS) signaling pathway. As a result, osteogenic-related gene expression was significantly enhanced after resveratrol treatment. ROS production in the cells was inhibited, while AMPK expression was upregulated by resveratrol [85].

To summarize, excessive ROS activates primarily adipogenic differentiation and suppresses osteogenic differentiation. In our experiments, the compound F1 caused oxidative stress, F2 caused an antioxidative state followed by short-term oxidative stress, and F3 did not cause changes in intracellular ROS. In all cases, neither adipogenic nor osteogenic differentiation was obtained. We hypothesized that the detected activation of genes of myogenic differentiation was not a consequence of ROS disbalance.

### 3.3. ROS and Autophagy in MSCs

The tight interactions between ROS and autophagy are manifested in two aspects: the induction of autophagy by oxidative stress and ROS reduction by autophagy. The superoxide anion radical is considered a critical cellular signaling molecule regulating autophagy [86]. Autophagy, in turn, serves to reduce oxidative damage and ROS levels through the removal of protein aggregates and damaged organelles such as mitochondria [87]. Depletion of autophagy in MSCs exacerbates oxidative-stress-induced MSC death [88]. The internal regulatory mechanisms of autophagy by ROS can be summarized as transcriptional and post-transcriptional regulation, which includes various molecular signal pathways such as ROS–FOXO3–LC3/BNIP3–autophagy, ROS–NRF2–P62–autophagy, ROS–HIF1–BNIP3/NIX–autophagy, and ROS–TIGAR–autophagy. Autophagy also may regulate ROS levels through several pathways such as the chaperone-mediated autophagy pathway, mitophagy pathway, and P62 delivery pathway [89].

Autophagy flux is considered a self-defensive process during the early stage of MSC injury, and this protective effect is abolished after prolonged oxidative exposure (several hours) [90]. In contrast, destructive autophagy is induced when it fails to neutralize excessive ROS [91].

In our experiments, short-term (within one hour) intracellular oxidative stress induced by compound F2 might induce self-defensive autophagy. Compound F3 did not affect ROS homeostasis. However, compound F1 caused prolonged oxidative stress, but did not induce autophagy. In general, we can assume that F2 caused autophagy due to fast short-term oxidative stress, and the subsequent antioxidant state was a consequence of autophagy.

### 3.4. The Role of pH and Potassium

A possible reason for the induction of autophagy by compound F2 may also be its alkalic character. Phosphonic acid is a medium-strength acid in the first stage and a weak acid in the second stage (*K*_1_ = 5.1·10^−2^, *K*_2_ = 1.8·10^−7^). A 200 μM solution of F2 contains 1 mM of phosphonate groups, which creates a pH of about 9 and may cause intracellular alkalosis. It is known that intracellular pH value influences MSC proliferation, differentiation, and paracrine activity [92]. Indeed, the activation of the autophagic pathway can be caused by alkaline stress [93].

To sum, we hypothesized that F2 induces autophagy due to intracellular alkalosis and/or short-term oxidative stress, followed by an antioxidative state.

The compound F2 contains potassium, and in the case of high concentration, 2 mM potassium enters the extracellular environment, and is comparable to its initial content. Recent studies confirm the significant role of potassium in the life of pluripotent stem cells [94]. Calcium-activated potassium channels have significant involvement in MSC differentiation, and could potentially enable novel tissue engineering approaches and therapies [95]. Calcium-dependent potassium channels control the proliferation of cardiac progenitor cells and bone-marrow-derived mesenchymal stem cells [96]. Adipose-derived stem cells can differentiate into pacemaker-like cells via overexpression of the SK4 calcium-dependent potassium channels gene [97]. Taking this into account, we cannot exclude the effect of potassium on the differentiation of mesenchymal stem cells.

### 3.5. Autophagy and Differentiation in MSCs

Recent studies have demonstrated that autophagy is necessary for differentiation processes in mesenchymal stem cells [92], especially towards the osteoblastic lineage [98]. Autophagy seems to be important in the control of osteogenic differentiation, and this seems to be related to the early mTOR inhibition and the late activation of the Akt/mTOR signaling axis [99]. However, conflicting results have been reported on whether rapamycin decreases or increases osteogenesis, according to the cell type [100,101], once again suggesting that no general statement can be made on the role of autophagy. Interestingly, autophagy induced the skeletal myogenic differentiation of human-tonsil-derived mesenchymal stem cells [33].

Thus, we hypothesize that F2-induced autophagy may result in the activation of genes of myogenic differentiation of MSCs.

### 3.6. Carbon Nanomaterials as Modulators of Differentiation in MSCs

Fullerenes have attractive outlooks in biology and medicine due to their unique mechanical, electrical, thermal, chemical, and optical properties [102]. Functionalization diminishes the toxicity of fullerenes and makes them water-soluble. Pristine fullerenes and their derivatives are considered as “drugs” (i.e., as inhibitors of human immunodeficiency virus protease and transcriptase), antiviral pharmaceuticals, radical scavengers, drug and gene carriers, photosensitizers, and contrast agents for magnetic resonance imaging [103,104,105]. In addition, fullerenes provide bacterial and viral inhibition, anticancer effects, and immunomodulation [106].

Carbon nanomaterials modulate the biological behavior of MSCs by regulating the expression of MAPK. Park et al. [107] found that 2D graphene regulates the cardiomyogenic differentiation of MSCs by enhancing the expression of extracellular matrix proteins and cellular FAK-Src-ERK/JNK signaling molecules, while 1D carbon nanotubes induced MSC differentiation towards cardiac progenitor cells in vitro and in vivo [108,109]. Yang et al. [110] found that the 0D fullerenol enhances the osteogenesis of human adipose-derived stem cells due to their great antioxidative capacity. The authors investigated the effects of C_60_ fullerene on the biological behavior of brown-adipose-derived stem cells, including survival, apoptosis, proliferation, and cardiomyogenic differentiation. C_60_ fullerene improved the MAPK expression level and stem cell survival, proliferation, and cardiomyogenesis. In addition, C_60_ fullerene improved the expression of cardiomyocyte-specific proteins (cTnT and alpha-sarcomeric actinin) and promoted gap junction formation among cells [36]. In our study, modification of C_60_ resulted in the induction of myogenic differentiation in MSCs. Thus, fullerenes can be considered regulators of stem cells differentiation in all possible variants, and targeted modification of their structure can be a promising way to modify their properties.

Limitations. Here, we studied only the expression of genes and proteins. Even though F2 fullerene stimulated the differentiation towards the myogenic lineage up to the late stages, a complete understanding of its differentiation potential can be achieved after studying the functionality and characteristics of myocytes. This topic may be a goal for further research.

## 4. Materials and Methods

### 4.1. Synthesis of Phosphonate Fullerene Derivatives

The C_60_ fullerene pentaphosphonic acid methyl ester (F1), C_60_ fullerene pentaphosphonic acid potassium salt (F2), and C_60_ fullerene pentaphosphonic acid 2-methoxyethyl ester (F3) were synthesized at the Institute of Problems of Chemical Physics of Russian Academy of Sciences (Chernogolovka, Russia). All the compounds were highly soluble in water and the culture medium. Compounds F1–F3 were synthesized using the reaction of chlorofullerene C_60_Cl_6_ and phosphites, as reported previously [39,111]. Compounds F1–F2 were characterized previously [111]. Spectral data for compound F3 are given below.

Compound F3 (Yield 25%)—NMR ^1^H (500 MHz, CDCl_3_, δ, ppm): 3.24–3.38 (m, 30H), 3.59–3.73 (m, 20H), 4.32–4.60 (m, 20H), 5.76 (d, 1H, *J* = 23.6 Hz).

NMR ^31^P (202 MHz, CDCl_3_, δ, ppm): 16.22–17.40 (m, 4P), 21.05–21.39 (m, 1P).

NMR ^13^C (125 MHz, CDCl_3_, δ, ppm): 54.54 (C_sp3 cage_-H), 56.97 (C_sp3 cage_-P), 57.64 (C_sp3 cage_-P), 58.06 (C_sp3 cage_-P), 58.69–58.82 (m, CH_3_), 59.16 (C_sp3 cage_-P), 60.46 (C_sp3 cage_-P), 61.72 (C_sp3 cage_-P), 66.94–67.37 (m, CH_2_), 71.37–71.70 (m, CH_2_), 138.70, 143.19, 143.33, 143.70, 143.88, 143.92, 144.32, 144.37, 144.58, 144.81, 145.22, 145.61, 145.85, 146.26, 146.67, 146.90, 146.97, 147.35, 148.07, 148.14, 148.24, 148.29, 148.39, 148.66, 148.70, 149.02, 149.45, 150.60.

ESI mass spectrum: *m*/*z* = 1705 ((M-H)^−^).

### 4.2. Adipose-Derived Mesenchymal Stem Cells

Four MSC cultures from the Research Centre for Medical Genetics cell culture collection were isolated from normal adipose tissue of breasts of patients with adenocarcinoma subjected to a surgical operation. To obtain stromal cells, minced adipose tissue was digested with collagenase as described previously [112]. The tissue samples were mechanically disrupted in Dulbecco’s Modified Eagle medium (DMEM) (Paneko, Moscow, Russia) containing 250 μg/mL gentamycin, 60 U/mL penicillin, and 60 U/mL streptomycin (Paneko, Moscow, Russia). The cells were dissociated by incubation with 0.04% collagenase (Sigma, St. Louis, MO, USA) in DMEM with 10% fetal bovine serum (FBS) (PAA Laboratories, Pashing, Austria) at 37 °C for 16 h. The cells were centrifuged at 200× *g* for 10 min, transferred into slide flasks, and cultivated at 37 °C in an AmnioMax C-100 Basal Medium (Gibco Products, Big Cabin, OK, USA) containing AmnioMax Supplement C-100, 20 mmol/L HEPES (Paneco, Moscow, Russia) and antibiotics. The cultures were split no more than four times before experiments. The fourth subculture was used for the experiments.

MSCs were characterized by standard markers using fluorescence-activated cell sorting (FACS). The surface proteins expression was studied by flow cytofluorimetry with the appropriate antibodies with the CyFlow device (Sysmex Partec, Görlitz, Germany). The obtained CD-markers profile was typical for MSCs: CD34–, CD45–, HLA-ABC+, HLA-DR–, CD44+, CD29+, CD49b low, CD54 low, CD90+, CD106–, CD105+, CD117– [113]. In addition, cells differentiated into adipocytes in the presence of inducers in a kit for adipogenic differentiation (MesenCult Adipogenic Differentiation Kit, STEMCELL Technologies, Vancouver, BC, Canada).

### 4.3. Culturing Cells with the Fullerenes

We examined the effect of water-soluble C_60_ fullerene derivatives F1, F2, and F3 in two concentrations on the expression of both genes and proteins. F1 was taken at concentrations of 398 nM and 170 μM; F2 was taken at concentrations of 456 nM and 195 µM; and F3 was taken at concentrations of 679 nM and 290 µM. The nanomolar concentrations were 427 times lower than the maximum nontoxic concentrations of the studied substances. MSCs were cultured with the fullerenes for 14 days. The culture medium was replaced by new portions every 4 days. The expression of differentiation markers was assessed at 7 and 14 days after the cultivation.

In addition, the cells were subjected to differentiation in the presence of inducers in a kit for myogenic differentiation (MyoCult Differentiation Kit, STEMCELL Technologies, Vancouver, BC, Canada).

Ethical approval for the use of the MSCs was obtained from the Regional Committees for Medical and Health Research Ethics (September 2016, approval #5).

### 4.4. MTT Assay

Cells were grown in a 96-well plate for 72 h. Cell viability was assessed with the 3-(4,5-dimethylthiazol-2-yl)-2,5-diphenyltetrazolium bromide (MTT) assay, as described previously [40,41]. The plates were analyzed at 550 nm with EnSpire Plate Reader (EnSpire Equipment, Turku, Finland).

### 4.5. Fluorescence Microscopy

Fluorescence microscopy was performed using an AxioScope A1 microscope (Carl Zeiss, Oberkochen, Germany) fluorescence microscope. The cells were fixed with 3.7% formaldehyde for 20 min at +4 °C and permeabilized with 0.1% Triton X-100 in PBS followed by washing and blocking with 1% albumin solution in PBS and incubated overnight with primary antibodies to beclin1 (sc48341), OPN (Sc-20788), osteocalcin (s390877) (Santa Cruz Biotechnology, Dallas, TX, USA), LC3 (NB100-2220), Myf5 (NBP1-19565), MYF6 (NBP1-55582), MYOD1 (NBP1-54153), and Myogenine (NBP1-95760) (NovusBio, Centennial, CO, USA) at +4 °C (1 µg/mL in PBS in the presence of 1% albumin). Next, after washing with PBS, the cells were incubated for 1 h with secondary antibodies m-IgGκ BP-FITC: sc-516140 (mouse IgGκ light chain binding protein) and mouse anti-rabbit IgG-FITC: sc-2359 (Santa Cruz Biotechnology, Dallas, TX, USA) at room temperature, washed in PBS and, if necessary, stained with DAPI.

### 4.6. Gene Expression by Real-Time PCR Assay

RNA was extracted from the cells using YellowSolve kits (Klonogen, St.-Petersburg, Russia) or Trizol reagent (Invitrogen, Carlsbad, CA, USA) according to the specified method (http://tools.lifetechnologies.com/content/sfs/manuals/trizol_reagent.pdf, accessed on 22 March 2021). Next, phenol-chloroform extraction and precipitation with chloroform and isoamyl alcohol (49:1) were performed. The RNA concentration was determined using a Quant-iT RiboGreen RNA reagent dye (MoBiTec, Göttingen, Germany) on a tablet reader (EnSpire Equipment, Turku, Finland), λ_em_ = 487 nm, λ_fl_ = 524 nm. According to the standard protocol, the reverse transcription reaction was performed using reagents from Sileks (Moscow, Russia).

PCR was performed using the specific primers (Syntol, Moscow, Russia) and SybrGreen intercalating dye on a StepOnePlus device (Applied Biosystems, Foster City, CA, USA). The primers used in the study are listed below as (F;R):

*TBP* (reference gene) (F: GCCCGAAACGCCGAATAT, R: CCGTGGTTCGTGGCTCTCT).

*MYOD1* (GGTCCCTCGCGCCCAAAAGAT; GTTCTCCCGCCTCTCCTAC);

*MYOG* (AGTGCACTGGAGTTCAGCG; TTCATCTGGGAAGGCCACAGA);

*MYF5* (CTGCCAGTTCTCACCTTCTGA; CGTCCCCAAATTCACCCTCG);

*MRF4* (AATCTTGAGGGTGCGGATTTC; CTCCTCCTTCCTTAGCCGTTA);

*BMP2* (ACTACCAGAAACGAGTGGGAA; CATCTGTTCTCGGAAAACCTGAA);

*BMP4* (TAGCAAGAGTGCCGTCATTCC; GCGCTCAGGATACTCAAGACC);

*RUNX2* (CCGTCTTCACAAATCCTCCCC; CCCGAGGTCCATCTACTGTAAC);

*SPP1* (CTCCATTGACTCGAACGACTC; GGTCTGCGAAACTTCTTAGAT);

*OCN* (CCCTCACACTCCTCGCCCTATT; AAGCCGATGTGGTCAGCCAACTCGT);

*LPL (*ACAAGAGAGAACCAGACTCCAA; GGTAGTTAAACTCCTCCTCC);

*AP2* (TGTGCAGAAATGGGATGGAAA; CAACGTCCCTTGGCTTATGCT);

*CEBPB* (CTTCAGCCCGTACCTGGAG; GGAGAGGAAGTCGTGGTGC);

*LC3* (AACATGAGCGAGTTGGTCAAG; GCTCGTAGATGTCCGCGAT);

*BECN1* (ACCTCAGCCGAAGACTGAAG; AACAGCGTTTGTAGTTCTGACA).

The reaction PCR mixture in the 25 µL volume consisted of 2.5 µL PCR buffer (700 mmol/L Tris-HCl, pH 8.6; 166 mmol/L ammonium sulfate, 35 mmol/L MgCl_2_, 2 µL 1.5 mmol/L dNTP solution; and 1 µL 30 pmol/L primer solution, cDNA. PCR conditions were selected individually for each pair of primers. After denaturation for 4 min at 95 °C, 40 amplification cycles were performed in the following order: 94 °C for 20 s, 56–62 °C for 30 s, 72 °C for 30 s, and 72 °C for 5 min.

The gene expressions were analyzed in several independent experiments in cells from different donors, and the results were processed using a calibration plot. The error was 2%.

### 4.7. Flow Cytometry Assay

According to the standard protocol, the protein expression was studied using the appropriate monoclonal antibodies (Santa Cruz Biotechnology, Dallas, TX, USA). Treated and blank cells were removed from the medium, washed with 1% albumin in PBS solution, fixed with 3.7% formaldehyde for 10 min at 37 °C, washed, and permeabilized in 90% methanol at –20 °C. Next, the cell suspension was incubated with primary antibodies (1 µg/mL) to beclin1 (sc48341), OPN (Sc-20788), osteocalcin (s390877), (Santa Cruz Biotechnology, Dallas, TX, USA), LC3 (NB100-2220), Myf5 (NBP1-19565), MYF6 (NBP1-55582), MYOD1 (NBP1-54153), and Myogenine (NBP1-95760) (NovusBio, Centennial, CO, USA) overnight at +4 °C (1 µg/mL in PBS in the presence of 1% albumin), and, if necessary, with secondary antibodies (mouse anti-rabbit IgG-FITC: sc-2359; anti-mouse: sc-516140 (Santa Cruz Biotechnology, Dallas, TX, USA)) for 1 h at room temperature in the dark, and analyzed with a Cyflow cytofluorometer (Sysmex Partec, Görlitz, Germany).

### 4.8. Statistics

Experiments were repeated in triplicate. In the FCA, the medians of the signal intensities were analyzed. Figures show the mean and standard deviation (SD). The significance of the observed differences was analyzed with the nonparametric Mann–Whitney *U-*test. The *p*-values < 0.005 were considered statistically significant, and are marked on figures with the “∗” sign. The data were analyzed with Excel, Microsoft Office (Microsoft, Redmond, WA, USA); Statistica 6.0 (Dell Round Rock, TX, USA); and StatGraphics (Statgraphics Technologies, The Plains, VA, USA).

## 5. Conclusions

We have shown that during prolonged culture of MSCs (7–14 days) in the presence of F2 fullerene, the cells were differentiated towards myogenic lineage. The expression of genes of transcription factors (*MYOD1*, *MYOG*, *MYF5*, and *MRF4*) that determine myogenic differentiation increased. The expression of genes of factors determining osteogenic differentiation (*BMP2*, *BMP4*, *RUNX2*, *SPP1*, and *OCN*) and adipogenic differentiation (*CEBPB*, *LPL*, and *AP2* (*FABP4*)) was reduced or did not change. One of the reasons for the myogenic differentiation effect of F2 may be the stimulation of autophagy in cells due to intracellular alkalosis and/or short-term intracellular oxidative stress. This finding opens new prospects for the use of newly synthesized fullerene derivatives.

## Figures and Tables

**Table 1:** Influence of the studied phosphonate fullerene derivatives on MSC differentiation lineage, intracellular ROS, and autophagy.

	F1	F2	F3
Chemical name	C_60_-pentadimethylphosphonate	C_60_-pentaphosphonate, potassium salt	C_60_-pentadimethoxyethylphosphonate
Chemical structure	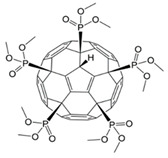	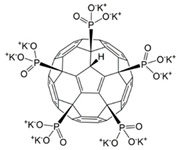	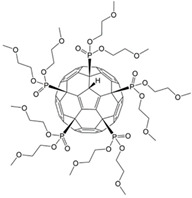
Maximal nontoxic concentration	175 µM	200 µM	295 µM
Intracellular ROS	A nonsignificant decrease in ROS during the first two hours, followed by oxidative stress	An increase in ROS during the first hour, followed by antioxidative state	No significant effect on intracellular ROS
Autophagy	No effect	Activates autophagy	No effect
Myogenic differentiation	Activation of the early stages of myogenic differentiation	Activation of the complete process of myogenic differentiation	Activation of the early stages of myogenic differentiation
Osteogenic differentiation	No effect	No effect	No effect
Adipogenic differentiation	No effect	No effect	No effect

## Data Availability

Not applicable.

## Appendix A

**Table A1:** The expression of genes and proteins of myogenic differentiation induced by F2 fullerene or the standard differentiation kit (a positive control) after 14 days of incubation; the gene RNA amount and protein amount were the mean values for three experiments related to the blank. In the blank experiments, cells were incubated without F2 or the differentiation kit.

Differentiation Stimulus	MYF5		MYOD		MYOG		MRF4	
	Gene	Protein	Gene	Protein	Gene	Protein	Gene	Protein
F2 (456 nM)	2.6	2.6	4.1	2.7	2.5	1.7	4.7	1.8
F2 (195 µM)	6.7	4.2	9.4	2.7	2.9	2.1	4.9	2.4
Positive control	1.2	1.1	3.9	2.6	4.1	3.1	5.1	2.7
